# Identifying reduced hearing in children who have developmental disabilities: Insights for inclusive research practices with electronic health records

**DOI:** 10.3389/fpsyg.2023.1134034

**Published:** 2023-03-15

**Authors:** Angela Yarnell Bonino, Deborah Mood

**Affiliations:** ^1^Department of Speech, Language, and Hearing Sciences, University of Colorado Boulder, Boulder, CO, United States; ^2^Department of Hearing and Speech Sciences, Vanderbilt University Medical Center, Nashville, TN, United States; ^3^Department of Pediatrics, University of Colorado Denver | Anschutz Medical Campus, Aurora, CO, United States

**Keywords:** developmental disabilities, audiology, children, big data, health care disparities, autism, Down syndrome, hearing

## Abstract

**Introduction:**

Recent advancements in big data analytics and the formation of large-scale clinical data repositories provide a unique opportunity to determine the current state of pediatric hearing health care for children who have developmental disabilities. Before answering unresolved questions about diagnostic practice, it is paramount to determine a standard and reliable method for identifying children who have reduced hearing because clinical management is affected by hearing status. The purpose of this study was to compare 5 different methods for identifying cases of reduced hearing from pure-tone thresholds based on developmental disability status.

**Methods:**

Using retrospective clinical data from 100,960 children (0–18 years), hearing status was determined for a total of 226,580 encounters from three clinical sites. 9% of the children had a diagnosis of intellectual disability, autism spectrum disorder, Down syndrome, or cerebral palsy.

**Results:**

Results revealed that encounters from children who have developmental disabilities were more likely to have insufficient data to allow hearing status to be determined. Moreover, methods with higher data demands (i.e., number of thresholds and ear-specific thresholds) resulted in fewer classifiable encounters. The average child age when hearing status was classified for the first time was older for children who have developmental disabilities than for children in the comparison group. Allowing thresholds to build up over multiple test sessions did result in more children who have developmental disabilities being classified than for single-encounter methods, but a meaningful decrease in child age at the time of classification was not seen for this strategy. Compared to the comparison group, children who have developmental disabilities were more likely to have reduced hearing that was stable over time, yet their hearing status was determined at older ages.

**Discussion:**

Results provide key guidance to researchers for how to determine hearing status in children for big data applications using electronic health records. Furthermore, several assessment disparities are spotlighted for children who have developmental disabilities that warrant further investigation.

## 1. Introduction

Approximately 15% of children in the United States have a developmental disability (Boyle et al., 2011): a group of conditions with onset in early childhood resulting in impairments in physical, language, learning, social, or behavior functioning across the lifespan. Moreover, many specific developmental disabilities are associated with a high risk of reduced hearing compared to the general population, including intellectual disabilities (ID; Meuwese-Jongejeugd et al., 2006; Hild et al., 2008; Erickson and Quick, 2017), Down syndrome (DS; e.g., Kreicher et al., 2018b), and cerebral palsy (CP; Reid et al., 2011; Weir et al., 2018). Despite the need for routine hearing monitoring and management of reduced hearing in children who have developmental disabilities (Bull and Committee on Genetics, 2011; The Joint Committee on Infant Hearing, 2019), there is limited evidence to guide clinical decisions for this population (for exception see: Greenberg et al., 1978; Thompson et al., 1979; Tharpe et al., 2001; Cupples et al., 2018; Trudeau et al., 2021).

Recent advancements in big data analytics and the formation of large-scale data repositories of electronic health records provide a unique opportunity to determine the current state of, and gaps in, clinical care for subpopulations of children who have developmental disabilities which will open new horizons for advancements in pediatric hearing health care. The Audiological and Genetic Database (AudGenDB) is currently the largest publicly available database of pediatric audiological data in the United States. As described by Pennington et al. (2020), AudGenDB is a HIPAA-compliant repository of clinical records from patients who received hearing health care at Children’s Hospital of Philadelphia, Vanderbilt University Medical Center, or Boston Children’s Hospital. AudGenDB has been used to characterize hearing profiles for children who have various medical conditions (Weir et al., 2016a,b, 2018; Kreicher et al., 2018a; Lee et al., 2020). Before we can answer unresolved questions about diagnostic practices from clinical data obtained at multiple sites, it is paramount — and the purpose of the present study — to establish an appropriate and reliable strategy for identifying children who have reduced hearing. The reason for this is because clinical management is expected to be different based on hearing status.

One approach would be to determine hearing status based on diagnosis codes (ICD-9/ICD-10) entered at the time of encounter for billing purposes (e.g., Dillard et al., 2020). However, this information is often not reliable in repositories derived from electronic health records, including AudGenDB,^1^ because there are substantial differences across clinical sites, and over time, in the use of hearing-related diagnosis codes. In the absence of reliable hearing-related billing information, published studies using AudGenDB have relied on threshold values in the audiogram record to determine hearing status (e.g., Weir et al., 2016a; Kreicher et al., 2018b). The use of the audiogram to identify cases of reduced hearing is aligned with the recognition of this assessment as the gold-standard diagnostic test starting at 4 to 6 months of age (American Speech-Language-Hearing Association, 2004; American Academy of Audiology, 2020).

One challenge with identifying cases of reduced hearing based on audiogram data is that children who have developmental disabilities are disproportionately at risk for having limited behavioral thresholds in their health record. Compared to typically-developing, age-matched controls, laboratory studies using clinical methods have confirmed that fewer behavioral thresholds are obtained for infants and children who have multiple conditions (Gans and Gans, 1993); 3- to 10-year-old children who have autism spectrum disorder (ASD; Tharpe et al., 2006); or 6- to 72-month-olds with DS (Greenberg et al., 1978). Similarly, retrospective clinical studies confirm that children who have developmental disabilities are at risk for not obtaining behavioral thresholds (Meagher et al., 2021; Trudeau et al., 2021), and thresholds were obtained from children who have DS at ages later than recommended (Nightengale et al., 2020). We posit that it is difficult to measure behavioral thresholds in children who have developmental disabilities because clinical methods are based on assumptions of typical child development. Thus, a high mismatch between a child’s developmental profile and the developmental demands of the behavioral testing method reduces the likelihood of collecting behavioral data. Because of these observations, research is needed to understand how algorithms used to identify cases of reduced hearing from audiogram data may affect the inclusion of children who have developmental disabilities.

A second challenge with using the audiogram to identify cases of reduced hearing is that there is not a universally accepted definition of reduced hearing based on threshold data. One parameter that is different across pediatric guidelines is which test frequencies should be measured (American Speech-Language-Hearing Association, 2004; Ontario Infant Hearing Program, 2019; American Academy of Audiology, 2020). Second, there is not a consistent definition for what value constitutes an elevated threshold, or whether one or multiple thresholds need to be elevated. It is often advocated that 15 dB HL should be used as the upper limit of typical hearing sensitivity in children (Northern and Downs, 2002; Moore et al., 2020). However, a specific definition of reduced hearing is not provided in the pediatric diagnostic guidelines from the two main professional organizations for audiologists in the United States (American Speech-Language-Hearing Association, 2004; American Academy of Audiology, 2020). Historically, a more conservative definition is used by World Health Organization (2016): > 25 dB HL. Similarly, to these inconsistencies, there is variability across epidemiological studies determining the prevalence of reduced hearing in the pediatric population (Lieu, 2017). Thus, these examples highlight that the field of audiology lacks consensus for how to identify cases of reduced hearing from audiometric data.

Given these potential challenges for identifying cases of reduced hearing in retrospective clinical data, the purpose of this methodology study was to determine the effectiveness of different classification methods of audiogram data based on developmental disability status. Encounters were classified as being from children who have a developmental disability if the child had a diagnosis of ID, ASD, DS, or CP in their health record. Encounters from children who did not have these diagnoses were used as the comparison group. Drawing on previous research studies and clinical guidelines, the present study evaluated five classification methods which varied based on the number of thresholds required to make the classification; the number of thresholds required to be elevated; and if data were obtained in a single or multiple encounters. For each method, reduced hearing was classified with a conservative or liberal criterion (> 25 or > 15 dB HL). The effectiveness of the methods and criterion was described based on two parameters: the proportion of encounters that could be classified and child age at time of classification. It was predicted that as data demands increased to determine hearing status, the proportion of classifiable encounters would worsen, and child age at the time of classification would increase. This pattern was expected to be magnified for children who have developmental disabilities because of limited audiogram data in the health record. By evaluating the effectiveness of these classification methods, results from this study were expected to (1) provide guidance on how to identify individual cases of reduced hearing from electronic health record data, and (2) shed light on potential disparities in behavioral hearing assessment practices for children who have ID, ASD, DS, or CP.

## 2. Materials and methods

This study was determined to be exempt from the Institution Review Boards at the University of Colorado Boulder and Vanderbilt University Medical Campus.

### 2.1. Patients and encounters

Electronic health records were accessed from 134,090 children (0–18 years) through AudGenDB. This publicly available, online database is HIPAA compliant, and all data are de-identified. AudGenDB contains audiologic testing, radiology reports, clinical genetic results, and medical health record data for patients who received audiological care at Children’s Hospital of Philadelphia, Vanderbilt University Medical Center, or Boston Children’s Hospital.

To construct the dataset for the present study, we first searched the database to identify all encounters that contained International Classification of Diseases 9th and 10th revision (ICD-9 and ICD-10) diagnosis codes from the encounter. To determine available encounters with audiogram data, the encounter was required to obtain at least one air conduction threshold with insert earphones, supra-aural headphones, or soundfield. Aided testing — pure-tone thresholds while wearing a listening device — and bone conduction thresholds were excluded. After filtering, the number of encounters that met these inclusion criteria was 226,580. Encounters were from a total of 100,960 children, with 81.2% receiving care at Children’s Hospital of Philadelphia, 10.8% at Boston Children’s Hospital, and 7.9% at Vanderbilt University Medical Center.

Using the ICD-9 and ICD-10 diagnosis codes, we identified 8,810 children who had a diagnosis of ID, ASD, DS, or CP, which hereafter we refer to collectively as children who have developmental disabilities. Specifically, children who have developmental disabilities had at least one of the ICD-9 or ICD-10 listed in Supplemental Table 1 in the child’s diagnosis or problem list record. Table 1 reports the number of children included for each developmental disability condition. If a child was identified as having a developmental disability, this flag was applied to all encounters for the child. Children who did not meet the criteria for developmental disabilities were classified as not having a developmental disability diagnosis — referred to as the comparison group in the present study.

**Table 1:** Overview of patients in the final sample separated by disability status.

Characteristic		Children with developmental disability diagnosis (*n* = 8,958)	Comparison group (*n* = 93,197)
Sex, *n* (%)	Male	6,172 (70.1%)	53,569 (58.1%)
	Female	2,637 (29.9%)	38,575 (41.9%)
Race, *n* (%)	White	5,251 (59.6%)	59,810 (64.9%)
	Black or African American	1754 (19.9%)	13,378 (14.5%)
	Asian	385 (4.4%)	2,908 (3.2%)
	Indigenous	20 (0.2%)	132 (0.1%)
	Other	1,400 (15.9%)	15,922 (17.3%)
Ethnicity, *n* (%)	Hispanic or Latino	861 (9.8%)	6,473 (7.0%)
	Not Hispanic or Latino	7,219 (81.9%)	67,148 (72.9%)
	Other	730 (8.3%)	18,529 (20.1%)
Developmental disability diagnosis, *n* (%)	Intellectual disability	1,459 (16.6%)	––
	Autism spectrum disorder	5,709 (64.8%)	––
	Cerebral palsy	1,179 (13.4%)	––
	Down syndrome	1881 (21.4%)	––
	Multiple diagnoses	1,229 (13.9%)	––
Encounters, mean (standard deviation)	Age of first encounter in the audiology clinic (year)	5.17 (4.24)	5.02 (4.15)
	Number of encounters	2.57 (2.92)	2.21 (2.45)

The number of children that were coded as having data that was missing, unknown, or declined to report was 7 for sex, 4,115, race, and 19,198 for ethnicity. Children with missing/unknown data for race or ethnicity data were counted in the “other” category. The mean child age for the first encounter and the number of encounters per child are reported based on the encounters in this dataset.

Table 1 provides an overview of the demographics for children included in the dataset stratified by developmental disability status. The number (*n*) and proportion of children for each demographic category are provided for the children examined in this study who had encounters with audiogram data. Children were classified based on their age, race, and ethnicity based on their disability status. Race was categorized into 5 groups: white, Black or African American, Asian, Indigenous (including Indian, Native Alaskan, American Indian, Native Hawaiian, and Pacific Islander), and other (including “other,” unknown, missing, or not revealed). Ethnicity was categorized into 3 groups: Hispanic, non-Hispanic, and other (including “other,” unknown, missing, or not revealed). Race and ethnicity data should be interpreted cautiously as there were differences in available categories across sites and over time.

### 2.2. Methods for determining hearing status

For each encounter with audiogram data, hearing status was classified into one of three mutually exclusive categories: insufficient data, typical hearing, or reduced hearing. This determination was based on the recorded air conduction thresholds at 500, 1,000, 2,000, and 4,000 Hz for each ear. Soundfield thresholds were only used if ear-specific data were not available. No attempts were made to classify the type or severity of the reduced hearing. An encounter was flagged as “reduced hearing” if either ear reached criteria for that method.

As detailed in Table 2, we built five methods to classify hearing status. In the naming convention used for these methods, **T** is the number of thresholds required, **E** is the number of encounters considered, and **C** is the threshold criteria for classifying reduced hearing. The subscript then further defines each of these variables for the method. Possible T values are 1, 8, or 3–4; possible E values are single encounter (S) or multiple encounters (M); possible C values are a single elevated threshold (T) or an elevated PTA.

**Table 2:** Description of the required data and criteria of the reduced hearing classification for each method.

Method	Required data	Definition of reduced hearing
T_1_ E_S_ C_T_	A minimum of 1 threshold is obtained. Soundfield data are permitted.	*Liberal:* A single threshold: > 15 dB HL for ear-specific testing; > 20 dB HL for soundfield testing; or > 25 dB HL for infants (< 12 months). *Conservative:* A single threshold >25 dB HL.
T_8_ E_S_ C_T_	For each ear, thresholds at 500, 1,000, 2,000, and 4,000 Hz are obtained.	*Liberal:* A single threshold >15 dB at the four required frequencies for either ear. Or if any additional frequencies were measured and were > 20 dB HL. *Conservative:* A single threshold >25 dB at the four required frequencies for either ear. Or if any additional frequencies were measured and were > 25 dB HL.
T_8_ E_M_ C_T_	For each ear, thresholds at 500, 1,000, 2,000, and 4,000 Hz are obtained. Data can be obtained over multiple visits.	*Liberal:* A single threshold >15 dB at the four required frequencies either ear. Or if any additional frequencies were measured and were > 20 dB HL. *Conservative:* A single threshold >25 dB at the four required frequencies for either ear. Or if any additional frequencies were measured and were > 25 dB HL.
T_3-4_ E_S_ C_PTA_	Thresholds at 500, 1000, and 2000 Hz are obtained. Soundfield data are permitted.	*Liberal:* The pure-tone average (PTA-4 or PTA-3) is: > 15 dB HL for ear-specific testing; > 20 dB HL for soundfield testing; or > 25 dB HL for infants (< 12 months). *Conservative:* The pure-tone average (PTA-4 or PTA-3) is >25 dB HL.
T_3-4_ E_M_ C_PTA_	Thresholds at 500, 1000, and 2000 Hz are obtained. Soundfield data are permitted. Data can be obtained over multiple visits.	*Liberal:* The pure-tone average (PTA-4 or PTA-3) is: > 15 dB HL for ear-specific testing; > 20 dB HL for soundfield testing; or > 25 dB HL for infants (< 12 months). *Conservative:* The pure-tone average (PTA-4 or PTA-3) is >25 dB HL.

Method T_1_ E_S_ C_T_, the least restrictive method, results in a reduced hearing identification if a single threshold is elevated and does not have a requirement for the number of thresholds obtained during the encounter. This is the method that has been used by other AudGenDB studies to determine hearing status (e.g., Kreicher et al., 2018b; Close et al., 2020). Method T_8_ E_S_ C_T_ aligns with best-practice clinical recommendations for determining hearing status (American Speech-Language-Hearing Association, 2004): requiring threshold data for all four frequencies (500, 1,000, 2,000, and 4,000 Hz) from both ears. Based on the approach commonly used in epidemiological studies (e.g., Mehra et al., 2009; Van Naarden Braun et al., 2015), Method T_3-4_ E_S_ C_PTA_ identifies reduced hearing based on an elevated four frequency PTA (PTA-4): the average of 500, 1,000, 2000 and 4,000 Hz. If data at 4000 Hz are unavailable, a PTA-3 (500, 1,000, and 2000 Hz) is permitted. Additionally, Method T_3-4_ E_S_ C_PTA_ allows for the use of soundfield data if ear-specific thresholds are unavailable. The flexibility for using a PTA-3 and soundfield data was included to replicate the methods used by other studies using data from AudGenDB (e.g., Kreicher et al., 2018b; Close et al., 2020) and may be particularly useful to promote the inclusion of encounters from children who are young and/or have developmental disabilities.

In contrast to the other methods, Methods T_8_ E_M_ C_T_ and T_3-4_ E_M_ C_PTA_ allow threshold data to build up over multiple encounters. The rationale for this approach is that audiologists may obtain an audiogram for a child over multiple encounters. Except for the use of multiple encounters, Methods T_8_ E_M_ C_T_ and T_3-4_ E_M_ C_PTA_ are identical to Methods T_8_ E_S_ C_T_ and T_3-4_ E_S_ C_PTA_, respectively. The algorithm for Methods T_8_ E_M_ C_T_ and T_3-4_ E_M_ C_PTA_ searches through audiograms in chronological order to obtain missing threshold values. Once a particular threshold is obtained, it is not replaced by subsequent data. No time limitation was placed on the delay between encounters.

For each method, reduced hearing was classified based on a liberal or conservative criterion (> 15 or > 25 dB HL). Replicating the methodology used by other AudGenDB studies, in the liberal approach reduced hearing was defined as a threshold or PTA of >15 dB HL if obtained under insert earphones or headphones; > 20 dB HL if obtained in the soundfield, or > 25 dB HL for infants (< 1 year). In the conservative approach, reduced hearing was defined as a threshold or PTA of >25 dB HL based on World Health Organization guidelines (2016), regardless of transducer or child age. Although the conservative criterion excludes cases classified in the slight severity range (16 to 25 dB HL), it is expected to reduce the risk of over-identifying reduced hearing in children who were clinically managed as having typical hearing sensitivity.

### 2.3. Statistical procedures

Prior to analysis, we used data cleaning techniques based on SQL-like code in R (Wickham et al., 2019; R Core Team, 2021) to manipulate the multiple comma separated value (CSV) files to extract relevant information about patients’ demographics, encounter history, and behavioral data. We conducted exploratory data analyses of potential inconsistencies in variable entry between the three clinical sites. Except for differences in billing practices related to diagnoses codes for reduced hearing, we did not detect relevant site differences. As a result, we did not control for the site in our data sampling techniques or analyses. We performed statistical analyses with R (version 3.5.2) and Python (version 3). Consistent with the purpose of this study, we focused on descriptive statistics to describe the method in relevant and meaningful terms to guide researchers in developing classification systems. We limited the use of hypothesis testing given the large number of samples and the likelihood of significant results for small effects. All hypothesis tests were prespecified to be conducted at the 0.05 significance level. In this paper, *N* is used to denote the number of encounters and *n* is used to denote the number of children. In analyses that consider differences across the four developmental disabilities, encounters from children who have multiple conditions are included under all diagnosed disabilities.

## 3. Results

The purpose of this study was to evaluate the effectiveness of the classification methods on two key parameters: proportion of encounters that could be classified and child age at time of classification. These parameters were computed for each method, with both a liberal and conservative criterion. Except for the general observation section below, all other analyses were stratified by developmental disability status. A supplemental analysis was conducted to examine the stability of classification for a subset of children who had multiple encounters in the dataset.

### 3.1. General observations for the full dataset

Results are provided in Table 3 and are grouped by criterion for all encounters with audiogram data (*N* = 226,580) without consideration for developmental disability status. For the full dataset, the number of encounters in which hearing status could be determined varied across methods. Because the inclusion criteria for the study required encounters to have at least one threshold in the record, all encounters could be classified for Method T_1_ E_S_ C_T_. The proportion of encounters where hearing status could be determined was 0.806 for Method T_3-4_ E_S_ C_PTA_ and 0.867 for Method T_3-4_ E_M_ C_PTA_. The lowest proportion of classified encounters was seen for Methods T_8_ E_S_ C_T_ and T_8_ E_M_ C_T_ at 0.622 and 0.658, respectively. This pattern of results supports our hypothesis that the proportion of classifiable encounters decreases with increased threshold requirements. Additionally, one-tailed *t*-tests for differences in proportions of identifiable encounters confirm that there is a significant benefit for allowing data to build up over multiple encounters: T_8_ E_M_ C_T_ > T_8_ E_S_ C_T_ (*t* = 25.78, *p* < 0.001), and T_3-4_ E_M_ C_PTA_ > T_3-4_ E_S_ C_PTA_ (*t* = 56.31, *p* < 0.001). Allowing information to build up over time resulted in the inclusion of 299 patients with Method T_8_ E_M_ C_T_ and 541 patients with Method T_3-4_ E_M_ C_PTA_ who had insufficient data to be classified with the companion, single-encounter method.

**Table 3:** Results are provided below for all encounters (*N* = 226,580) in the database without the consideration of developmental disability status.

		Method T_1_ E_S_ C_T_	Method T_8_ E_S_ C_T_	Method T_8_ E_M_ C_T_	Method T_3-4_ E_S_ C_PTA_	Method T_3-4_ E_M_ C_PTA_
Determining hearing status	Number of classifiable encounters	226,580	140,954	149,079	182,709	196,531
	Proportion of classifiable encounters	1	0.622	0.658	0.806	0.867
	Age at hearing status determination, [95% CI], (*n*)	5.03 [5.01; 5.06] (100960)	6.80 [6.77; 6.83] (65850)	6.79 [6.76; 6.82] (66149)	5.36 [5.34; 5.39] (88222)	5.35 [5.32; 5.38] (88763)
Classification of reduced hearing with liberal criterion	Number of encounters with reduced hearing	134,503	87,440	95,583	89,154	101,785
	Proportion of encounters with reduced hearing	0.594	0.620	0.641	0.488	0.518
	Age at reduced hearing classification, [95% CI], (*n*)	5.60 [5.56; 5.63] (56027)	7.33 [7.28; 7.37] (35648)	7.30 [7.26; 7.35] (36015)	6.16 [6.11; 6.20] (38792)	6.10 [6.05; 6.14] (38553)
	Proportion of patients with a classification change, (*n*)	0.294 (47893)	0.229 (28528)	0.212 (29829)	0.282 (38224)	0.248 (40620)
Classification of reduced hearing with conservative criterion	Number of encounters with reduced hearing	102,929	67,955	75,663	56,459	66,301
	Proportion of encounters with reduced hearing	0.454	0.482	0.508	0.309	0.337
	Age at reduced hearing classification, [95% CI], (*n*)	5.62 [5.58; 5.67] (41895)	7.90 [7.85; 7.96] (25774)	7.87 [7.81; 7.92] (26119)	6.42 [6.36; 6.48] (23563)	6.35 [6.29; 6.41] (24127)
	Proportion of patients with a classification change, (*n*)	0.308 (47893)	0.226 (28528)	0.212 (29829)	0.213 (38224)	0.189 (40620)

For each method, the number and proportion of encounters in which hearing status was determined or classified as having reduced hearing are provided. Reduced hearing data are separated by criterion (liberal versus conservative). Age at hearing status determination is the mean child age of the first encounter in which hearing status could be determined. Age at reduced hearing classification is the mean child age of the first encounter classified as “reduced hearing” for those children with at least one encounter that was classified as having reduced hearing. For each method, the proportion of patients who had a change in their hearing status classification over time are provided. This estimate of the stability of the hearing classification (“typical hearing” versus “reduced hearing”) was computed by classifying two selected encounters for patients who had multiple encounters (*n* = 47,893). Some variables provide a 95% confidence interval (CI) of the mean in brackets and the sample size in terms of the number of patients (*n*) in parentheses.

Age at hearing status determination is the mean child age for the first encounter in which hearing status could be determined for each patient. Mean child age for hearing status determination was younger for Method T_1_ E_S_ C_T_ than all other methods. Relative to Method T_1_ E_S_ C_T_, the delay in age at hearing status determination was 0.3 years for Methods T_3-4_ E_S_ C_PTA_ and T_3-4_ E_M_ C_PTA_, and 1.8 years for Methods T_8_ E_S_ C_T_ and T_8_ E_M_ C_T_. These results suggest that child age was affected by the number of required thresholds. Inconsistent with our hypothesis, there was no clinically meaningful reduction of child age if threshold data were allowed to build up over multiple encounters. Collectively, these results reveal a cost associated with methods that require more threshold data for big data applications: a delay in age for being able to determine hearing status that cannot be offset by allowing data to build up over multiple encounters.

### 3.2. Number of classified encounters

**Figure 1:**
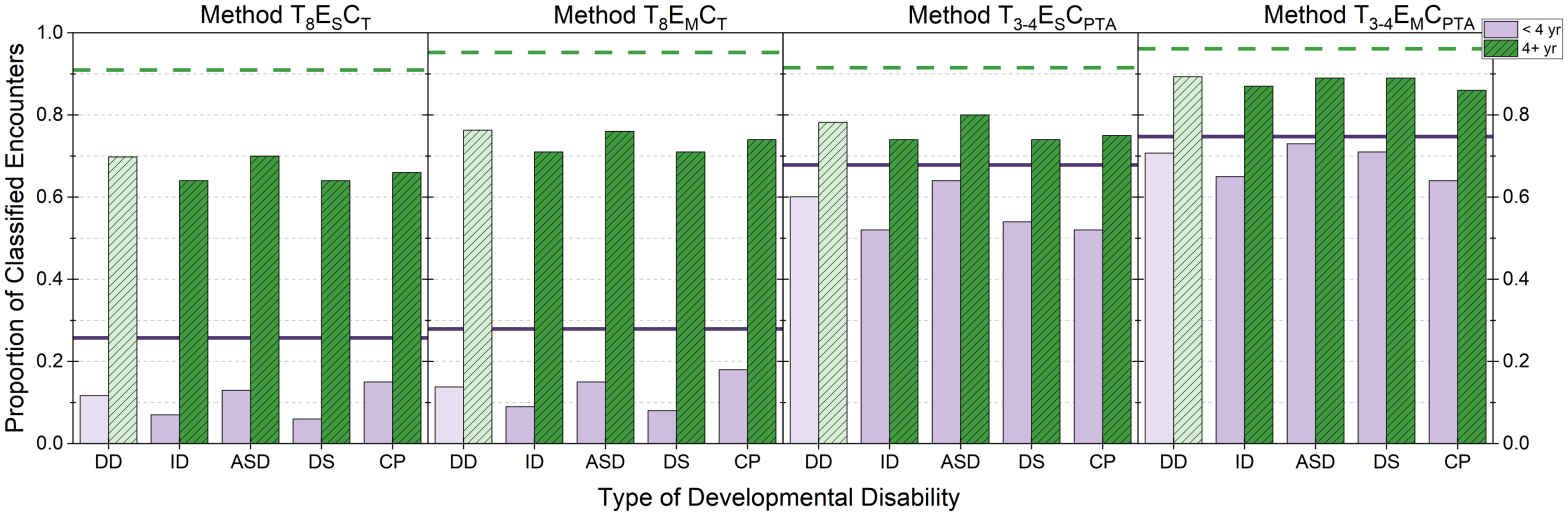
Proportion of classified encounters are shown by developmental disability diagnosis. Each panel represents a different method for classifying hearing status. Within a panel, data for children who have any of the developmental disability (DD) diagnosis (lighter scaling) are provided, followed by data for each diagnosis (ID, ASD, DS, and CP). For each diagnosis, encounters are separated based on child age at the time of encounter: < 4 years (purple, solid) and ≥ 4 years (green, hashed). For reference, the horizontal lines show the proportion of encounters classified for children in the comparison group who were < 4 years (purple, solid) or ≥ 4 years (green, dashed). Data from Method T_1_ E_S_ C_T_ are not shown because the proportion is 1.0 for all groups. For children <4 years, the number of encounters was 84,160 for the comparison group; 9,194 for DD; 990 for ID; 5,985 for ASD; 2,128 for DS; and 1,205 for CP. For children ≥4 years, the number of encounters was 119,739 for the comparison group; 13,417 for DD; 3,361 for ID; 6,723 for ASD; 4,849 for DS; and 1,783 for CP.

To evaluate the effects of developmental disability status on the ability to classify hearing status, Figure 1 shows the proportion of encounters that could be classified for each method. Each panel represents one method for classifying hearing status. Within a panel, the first two columns provide data for children who have any of the developmental disability (DD) diagnosis (lighter scaling), then data for each developmental disability diagnosis are provided (ID, ASD, DS, and CP). For each disability type, encounters were dichotomized into two age groups: < 4 years (purple, solid) or ≥ 4 years (green, hashed). The rationale for this age split is because by 4 years children can complete a full audiogram in a single clinical encounter (Barr, 1955; Nielsen and Olsen, 1997). The horizontal lines in each panel provide the proportion of encounters that could be classified from children in the comparison group. The purple (solid) line is an age of encounter of <4 years, whereas the green (dashed) line is an age of encounter of ≥4 years.

Figure 1 shows clear differences in the proportion of classifiable encounters based on developmental disability status and age at the time of encounter. Consistent with clinical literature (Barr, 1955; Nielsen and Olsen, 1997), for children ≥4 years without a developmental diagnosis, the proportion of classified encounters was 0.909 to 0.961 across the methods. In contrast to this group, differences across the classification methods were seen for both age groups in children who have developmental disabilities. However, the pattern of results across methods largely mirrors those reported above for the full dataset.

There are three trends that warrant further considerations. First, for children who have developmental disabilities, Methods T_3-4_ E_S_ C_PTA_ and T_3-4_ E_M_ C_PTA_ result in a higher proportion of classifiable encounters than Methods T_8_ E_S_ C_T_ and T_8_ E_M_ C_T_. This difference was particularly sizable for encounters from children <4 years. This pattern indicates that audiograms from young children or children who have developmental disabilities are likely to contain limited thresholds and/or soundfield results.

Second, there appears to be a greater benefit for allowing data to build up over encounters for children who have developmental disabilities than for children in the comparison group. However, the pattern of benefit for allowing data to build up over encounters appears to be relatively similar across the four different developmental disabilities. The largest benefit for allowing information to build up over time was seen for Method T_3-4_ E_M_ C_PTA_. Method T_3-4_ E_M_ C_PTA_ results in the inclusion of an additional 101 children who have developmental disabilities that could not be classified with Method T_3-4_ E_S_ C_PTA_.

The final observation is that, across all methods and regardless of child age, encounters from children who have developmental disabilities had lower rates of classifiable encounters than the comparison group. To evaluate this trend, the average proportion of classified encounters across all five methods was calculated based on developmental disability status. Encounters were not disaggregated by child age for this analysis. The proportion of classified encounters was 0.817 for children who have developmental disabilities (*N* = 22,621) and 0.873 for children in the comparison group (*N* = 203,959). This difference indicates that encounters are more likely to result in an incomplete audiogram for children who have developmental disabilities than for children in the comparison group. To further explore potential differences across the four developmental disability diagnoses, the proportion of classifiable encounters was computed for each subgroup. The proportion of classified encounters was 0.817 for ID (*N* = 4,352); 0.812 for ASD (*N* = 12,712); 0.834 for DS (*N* = 6,980); and 0.769 for CP (*N* = 2,991). This finding suggests that encounters from children who have CP have a lower classification rate than seen for encounters from children who have ID, ASD, or DS.

### 3.3. Child age at hearing status determination

Table 4 provides the mean and 95% confidence intervals for child age (in years) at the time of the first encounter with audiogram data at which hearing status could be determined for each method based on developmental disability status, as well as for each developmental diagnosis. The average age provided for determining hearing status for Method T_1_ E_S_ C_T_ reflects the first encounter in the clinic where behavioral threshold (s) were obtained. The mean age of first encounter in which behavioral testing is attempted is 1.9 months later for children who have developmental disabilities than for children in the comparison group. Moreover, mean age of hearing status determination is different across the four subgroups: ASD < CP < DS < ID. As shown in Table 4, the 95% confidence intervals for mean child age for Method T_1_ E_S_ C_T_ do not overlap with the mean child age for any of the subgroups. Consistent with these findings, hearing status was determined at older ages for the remaining methods for children who have developmental disabilities than for children in the comparison group. The one exception is children who have ASD. These results should be interpreted cautiously because it may be that this developmental profile affects the timeline for the referral to audiology as well as the ability to collect behavioral data. Moreover, the true age of onset of reduced hearing is likely different across the developmental disability subgroups.

**Table 4:** For each method, the average age (in years) is provided for the ability at determining hearing status by developmental status.

Developmental Status	Method T_1_ E_S_ C_T_	Method T_8_ E_S_ C_T_	Method T_8_ E_M_ C_T_	Method T_3-4_ E_S_ C_PTA_	Method T_3-4_ E_M_ C_PTA_
No diagnosis (comparison group)	5.02 [4.99; 5.05] (92150)	6.74 [6.71; 6.77] (61210)	6.73 [6.70; 6.76] (61455)	5.34 [5.32; 5.37] (80998)	5.33 [5.30; 5.36] (81438)
Developmental disabilities	5.17 [5.09; 5.26] (8810)	7.66 [7.54; 7.78] (4640)	7.63 [7.51; 7.75] (4694)	5.58 [5.48; 5.68] (7224)	5.55 [5.45; 5.65] (7325)
Intellectual disability	7.79 [7.53; 8.04] (1459)	9.82 [9.55; 10.09] (907)	9.80 [9.53; 10.07] (918)	8.27 [8.00; 8.55] (1180)	8.24 [7.97; 8.51] (1197)
Autism spectrum disorder	4.69 [4.59; 4.78] (5709)	6.97 [6.83; 7.11] (2756)	6.96 [6.82; 7.11] (2781)	4.94 [4.83; 5.05] (4720)	4.92 [4.81; 5.03] (4784)
Down syndrome	6.06 [5.85; 6.28] (1881)	8.98 [8.74; 9.22] (1125)	8.91 [8.67; 9.15] (1141)	6.78 [6.54; 7.03] (1537)	6.73 [6.49; 6.97] (1557)
Cerebral palsy	5.65 [5.38; 5.92] (1179)	7.97 [7.60; 8.34] (614)	7.92 [7.55; 8.28] (629)	6.23 [5.91; 6.55] (892)	6.20 [5.89; 6.52] (912)

The 95% confidence interval is reported in brackets. The number of children in each subgroup (*n*) is reported in parentheses. Recall that the average age provided for determining hearing status for Method T_1_ E_S_ C_T_ reflects the first encounter in the clinic where behavioral threshold (s) were obtained.

To further explore the difference in child age across the methods, while considering differences for when behavioral testing was first attempted, the gap in age was calculated between the three single-encounter methods. The age gap for Method T_8_ E_S_ C_T_ and Method T_3-4_ E_S_ C_PTA_ was calculated relative to Method T_1_ E_S_ C_T_. The calculated age gaps were larger for children who have developmental disabilities than for children in the comparison group. For example, the difference between Method T_1_ E_S_ C_T_ and Method T_8_ E_S_ C_T_ was 2.5 years for children who have developmental disabilities and 1.7 years for children in the comparison group. With the group of children who have developmental disabilities, this difference was 2.0 years for children who have ID, 2.3 years for children who have ASD, 2.9 years for children who have DS, and 2.3 years for children who have CP. These values should be interpreted cautiously because only 53% of children who have developmental disabilities have sufficient data in their record to allow classification for the two methods: 62, 48, 60, and 52% for children who have ID, ASD, DS, and CP, respectively. In contrast, 66% of children in the comparison group were able to be classified for both methods.

The final comparison of interest was to examine the difference between single- and multiple-encounter method pairs (T_8_ E_S_ C_T_ and T_8_ E_M_ C_T_; T_3-4_ E_S_ C_PTA_ and T_3-4_ E_M_ C_PTA_) to see if allowing data to build up resulted in an earlier age at the time of hearing status determination. Across all subgroups of children, the mean age of children at the time of classification is younger for multiple-encounter methods than single-encounter methods. However, as shown in Table 4, the value for the multiple-encounter method falls well within the 95% confidence interval estimate for the single-encounter, companion method for all subgroups of children. Thus, allowing data to build up over multiple test sessions does not result in a meaningful decrease in child age at the time of hearing status determination for children who have developmental disabilities.

### 3.4. Supplemental analysis: Stability of hearing status classification over time

One potential concern is that stability of hearing status may be variable across classification methods and developmental disability status. To evaluate the stability of each method, children were identified in the dataset who had at least two encounters with audiogram data. Multiple encounters were available from 47,893 patients in the dataset, of which 9.6% (*n* = 4,589) had a developmental disability. For each classification method, we compared the patient’s hearing status from the first classifiable encounter to a randomly selected classifiable encounter from a later time point. For each method, the proportion of classification change was computed: a change in the hearing status (i.e., “typical hearing” or “reduced hearing”) between the two encounters, without consideration for directionality. For each classification method, Supplemental Table 2 (liberal criterion) and Supplemental Table 3 (conservative criterion) provide the proportion of classification change, proportion of children who have reduced hearing, and sample size (*n*) by developmental disability status. Results from the build-up methods should be interpreted with caution because it is possible that the same thresholds could have been used to determine hearing status for the two encounters. Across all methods and criteria, the rate of change in classification was lower for children who had developmental disabilities than for children in the comparison group.

**Figure 2:**
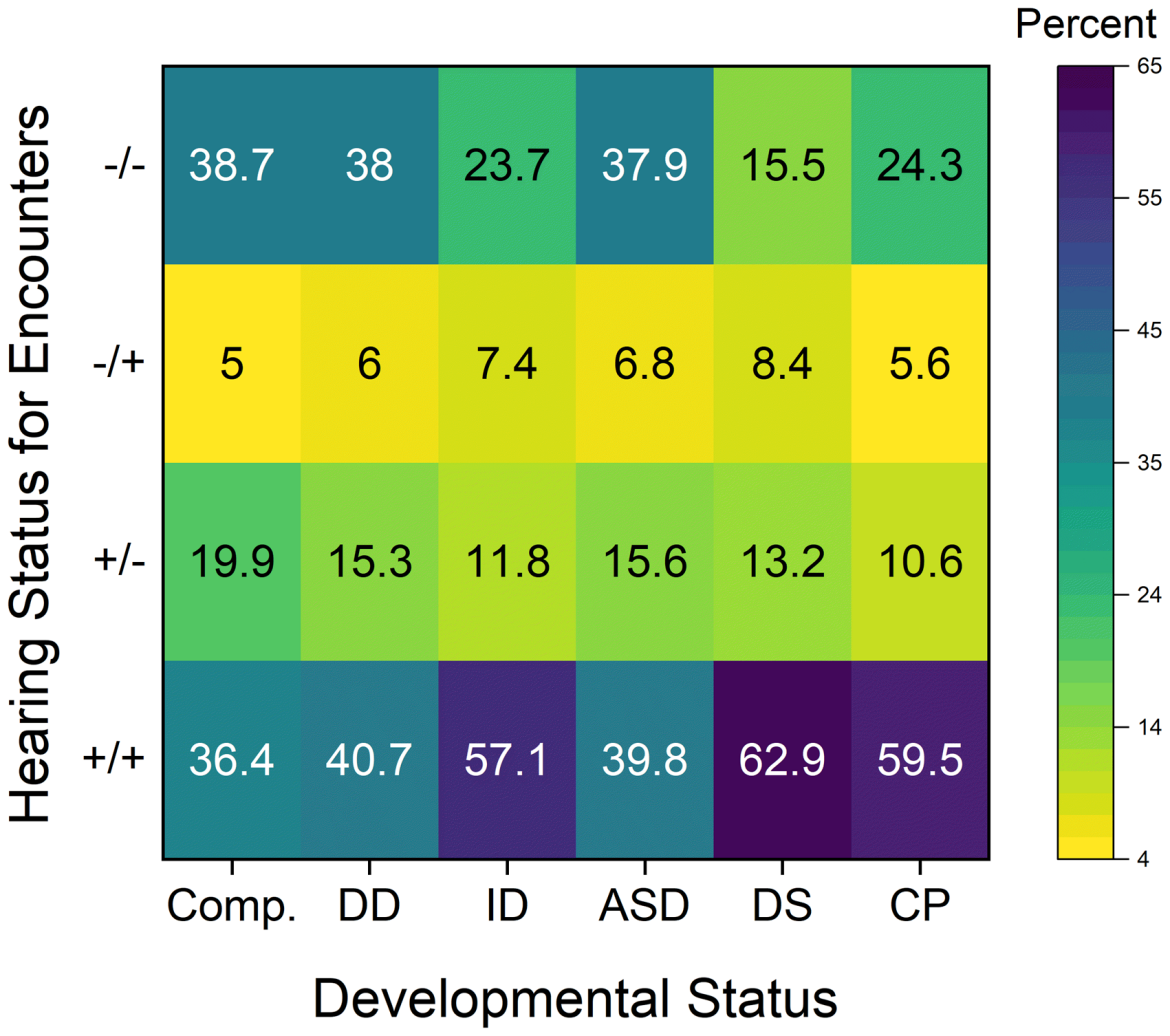
Across classification methods and criteria, the percentage of patients with a particular hearing classification combination over the two encounters are shown based on developmental status. Hearing classification categories were: “reduced hearing” classification for both encounters (“+/+”); “typical hearing” classification for both encounters (“−/−”); “reduced hearing” classification at the first encounter and “typical hearing” classification at the second encounter (“+/−”); or “typical hearing” classification at the first encounter and “reduced hearing” classification at the second encounter (“−/+”). Patients were grouped based on their developmental status (comparison group (“Comp.”) or developmental disabilities (DD), and then children who have developmental disabilities were further grouped based on their type of diagnosis. The colormap provides a visual representation for the percentage value in each box.

To further examine the stability of hearing classification by disability type, the combination of the classification from the two encounters was compared. Patients were assigned to one of four categories: “reduced hearing” classification for both encounters (“+/+”); “typical hearing” classification for both encounters (“−/−”); “reduced hearing” classification at the first encounter and “typical hearing” classification at the second encounter (“+/−”); or “typical hearing” classification at the first encounter and “reduced hearing” classification at the second encounter (“−/+”). Collapsing across the classification methods and criteria, Figure 2 provides the percentage of occurrence for each category based on developmental status. The colormap provides a visual representation for the percentage value in each box. Across all membership groups, only 5 to 8% of patients had instability because their classification shifted from “typical hearing” to “reduced hearing.” In contrast, most changes in classification were in the opposite direction, although children who have developmental disabilities were less likely to have this shift than children in the comparison group. Specifically, whereas 20% of patients in the comparison group had a shift from “reduced hearing” to “typical hearing” (+/−) across the two encounters, this category only described 10.6% (CP) to 15.6% (ASD) of encounters for the developmental disability subgroups. The prevalence of a stable “reduced hearing” classification (+/+) was substantially higher in children who have developmental disabilities than for children in the comparison group (DS > CP > ID > ASD). Thus, children who have developmental disabilities – especially those who have ID, DS, and CP – are at high risk for having reduced hearing that persists over both encounters.

## 4. Discussion

The approach used in this methodology study was to use a large, publicly available database of audiological records from children to evaluate five different methods for classifying hearing status (“typical hearing” versus “reduced hearing”) based on pure-tone thresholds. The effectiveness of each method was evaluated in terms of the proportion of encounters that could be classified and child age at the time of classification. A follow-up analysis described the stability of the classification over time for a subset of patients with multiple encounters. Results from this study confirm differences for these parameters across methods and based on developmental disability status. Findings from this study will guide researchers in how to identify cases of reduced hearing in retrospective clinical data. Moreover, results from the present study provide compelling evidence that the approach of using clinical data is a fruitful one for evaluating potential differences in assessment practices based on the developmental profile of children. Observed differences in the present study suggest that children who have a diagnosis of ID, DS, or CP may be particularly vulnerable to disparities in care.

### 4.1. Insights for researchers using electronic health records

Leveraging data in electronic health records provides a unique opportunity to accelerate the pace of pediatric research in the areas of audiology and developmental disabilities. This approach can be used to determine disparities and inequities in pediatric hearing health care, as well as determine prevalence of reduced hearing in childhood for specific developmental profiles. However, because hearing-related billing codes may not reflect actual hearing status, care must be used in the extraction and interpretation of raw clinical data to determine hearing status. Furthermore, as discussed below, the audiogram parameters used to identify cases of reduced hearing can disproportionately exclude children who have developmental disabilities in the final dataset. Results from this study can guide researchers in determining reduced hearing criteria which is inclusive of children who have developmental disabilities, and therefore, allowing representation of this population when conducting research to guide clinical decisions. These considerations will become important to guard against bias if precision medicine algorithms are incorporated into standard hearing health care in the future. These considerations are also important during the creation of large-scale repositories in response to open science initiatives to accelerate biomedical discoveries. To date, publicly available datasets or data hubs of individuals who have developmental disabilities often contain no hearing data; those that do are limited to hearing-related ICD codes. Results from the current study indicate there is need to capture raw clinical data to accurately understand the co-occurrence of reduced hearing in children who have developmental disabilities.

#### 4.1.1. Guidance for how to identify cases of reduced hearing from audiogram data

Results from this study highlight the need for careful consideration across multiple parameters to identify children who have reduced hearing based on audiogram data. As classification methods required more threshold data, there was a higher likelihood of encounters having insufficient data for classification, thereby resulting in delays in child age at the time of hearing status determination. One surprising finding is that allowing information to build up over multiple encounters did not substantially offset the proportion of unclassifiable encounters for methods with high threshold requirements. This finding contrasts with the widely accepted clinical perspective that an audiogram can be successfully obtained over multiple test sessions. Compared to the companion, single-encounter method, methods that allowed for data to build up over multiple encounters did not meaningfully reduce child age at the time of classification. However, these methods did result in statistically significant, albeit limited, improvement in the proportion of encounters that could be classified. The most noticeable improvement was seen for classifying encounters from children who have developmental disabilities: resulting in the inclusion of an additional 54 children for Method T_8_ E_M_ C_T_ and 101 children for Method T_3-4_ E_M_ C_PTA_ relative to the companion, single-encounter method. The implication for big data projects is that, depending on the research question, this somewhat limited increase in available patients, representing <1% of sample, may not outweigh the risk of potential shifts in hearing thresholds across encounters.

Benefits were seen for classifying reduced hearing based on a PTA rather than a single elevated threshold. For example, hearing classification was more stable for Method T_3-4_ E_S_ C_PTA_ than for Method T_1_ E_S_ C_T_ for patients with multiple audiograms. However, the occurrence of reduced hearing was also lower for Method T_3-4_ E_S_ C_PTA_ than for Method T_1_ E_S_ C_T_. Although using a PTA criterion may guard against the inclusion of some cases of transient reduced hearing, it is at the risk of excluding certain configurations. Another limitation of using a PTA criterion is that it requires a high number of thresholds to be collected. In the present study, we allowed for a PTA-3 to provide flexibility if data were not obtained at 4000 Hz. A PTA-3 was used in a limited number of encounters (e.g., 4.1% of encounters for Method T_3-4_ E_M_ C_PTA_ in the full dataset).

The inclusion of soundfield data resulted in more encounters being classifiable than for methods that required ear-specific thresholds. Although it is feasible and recommended to obtain ear-specific thresholds starting in infancy (Widen et al., 2000), audiologists routinely use soundfield testing. Of all encounters in the dataset, 24.3% had thresholds obtained in the soundfield. Furthermore, soundfield testing was used nearly twice as often in encounters from children who have developmental disabilities than for encounters from children in the comparison group. Based on our study, research applications that exclude threshold data obtained in the soundfield will exclude children who have developmental disabilities at a higher rate than children in the comparison group. The requirement of ear-specific thresholds also biases the sample to older children and children who have reduced hearing. This may be a byproduct of clinicians performing a full audiogram with ear-specific thresholds when reduced hearing is suspected. Careful consideration of the use of soundfield data, and how it relates to the inclusion of children who have developmental disabilities, is warranted in big data applications.

Lastly, differences in the occurrence of reduced hearing were seen based on the use of a conservative or liberal criterion. Previous epidemiological work suggests that slight reduced hearing (16 to 25 dB HL) reflects a large population of childhood reduced hearing cases (Su and Chan, 2017; Wang et al., 2019). Specifically, meta-analysis results from Wang et al. (2019) indicate that the prevalence of childhood reduced hearing is 13% for a criterion of >15 dB HL, and 2% for a criterion of >25 dB HL. In the present study, more encounters were classified as having reduced hearing with the liberal criterion (> 15 dB HL) than the conservative criterion (> 25 dB HL) by a factor of 1.3 to 1.6 across the classification methods in the full dataset. Additionally, the liberal criterion was less stable than the conservative criterion. It is not clear how often instability for the liberal criterion reflects true changes in hearing status, or differences in testing procedures between encounters and care is warranted when selecting the cut off criterion for typical hearing. Future research is needed to establish evidence-based recommendations for the cutoff for typical hearing in children who have developmental disabilities, as thresholds classified as “slight” or “mild” severity levels of reduced hearing have been associated with increased behavioral problems and reading difficulties in children who are typically developing (Yoshinaga-Itano et al., 2008; le Clercq et al., 2020; Walker et al., 2020). Researchers are advised to select a cut-off criterion that is appropriate for the population of interest and their research questions while considering the constraints of the protocol used to collect the behavioral thresholds.

#### 4.1.2. Considerations for the use of AudGenDB

Based on our work with AudGenDB, we have confidence that the database can be used to address unresolved questions about hearing profiles in children who have particular conditions or to examine patterns of diagnostic clinical care. However, this database is not well constructed to address questions about interventions for reduced hearing. In addition to known challenges with AudGenDB (Pennington et al., 2020), we identified other issues and provide a summary here to assist other researchers. In the audiogram record, we determined that the test method (e.g., visual reinforced audiometry) and transducer type were not reliable variables due to coding inconsistencies. Careful review of the data is needed to identify thresholds associated with either soundfield or aided testing as multiple codes are used. Missing data were commonly seen in the billing record. In our screening of 312,013 encounters with audiogram data, 7.0, 15.1, and 91.3% were missing diagnosis (ICD9CM/10), problem list (ICD9CM/10), or procedural codes (CPT4/HCPCS), respectively. Only 2.0% of encounters included all three code types; 8.5% of encounters contained both diagnosis and procedural codes. For studies evaluating different groups of children based on the diagnosis record, care must be exercised in the construction of the dataset to address the issue of missing data. Additionally, because of the extent of missing procedural codes it is difficult to implement data screening procedures that examine the completeness of the database by comparing clinical data to the billing code record.

### 4.2. Clinical implications: Disparities in assessment practices for children who have developmental disabilities and future directions

In addition to extending our knowledge of the appropriateness of different methods for identifying reduced hearing cases in applications using big data analytics, results from this study have important implications for clinical care of children who have developmental disabilities. Results in the present study provide clear evidence that pediatric audiologists have a difficult time obtaining a complete audiogram with current clinical methods in children who have developmental disabilities. About 28% of children who have developmental disabilities in AudGenDB had no audiogram data, whereas 23% of children in the comparison group had no audiogram data. When behavioral thresholds were recorded, they were more likely to be in the soundfield, rather than ear-specific thresholds, for children who have developmental disabilities than for the comparison group. Over reliance on soundfield testing means that children who have developmental disabilities are at risk for undiagnosed reduced hearing that is unilateral. Results from the present study also question the common clinical perception that an audiogram is successfully obtained over multiple test sessions. Collectively, these observations suggest that children who have developmental disabilities experience disparities in their access to the gold-standard assessment of hearing. Consequently, certain hearing profiles are likely at risk for being undiagnosed in children who have developmental disabilities (e.g., slight or mild, high frequency, and unilateral hearing profiles). This issue may be somewhat mitigated by the use of physiologic measures (e.g., auditory brainstem response and otoacoustic emissions), but these procedures are prone to missing some of the above hearing profiles and may require sedation. Challenges in adhering to recommended pediatric audiology diagnostic practices seen here and for other studies (Findlen and Schuller, 2020; Nightengale et al., 2020; Trudeau et al., 2021), suggests that there is a need for future research and clinical guidance to ensure equitable access for children who have developmental disabilities to a comprehensive hearing assessment in a timely manner.

Results from the present study suggest that certain developmental profiles are at more risk than others for providing limited behavioral threshold data. Encounters from children diagnosed with ASD generally had a higher rate of classification than those encounters from children who have ID, DS, or CP. Children who have CP were more likely to have encounters that could not be classified than the other disability subgroups. Additionally, children who have DS had larger age gaps across the different classification methods than children who have other developmental disabilities. These observations may reflect difficulty to collect a complete audiogram because of developmental misalignment between the child and task. Alternatively, audiologists may be prioritizing other types of clinical data in the management of children based on their developmental disability diagnosis. However, Trudeau et al. (2021) recently reported significant delays for making a referral for auditory brainstem response testing in children who have developmental disabilities when behavioral thresholds were not successfully obtained. The average delay was 9 months for children who have ASD; 22 months for children who have DS; and 20 months for children who have CP. Further research is planned to better understand the developmental profiles of children at greatest risk for having no or limited pure-tone threshold data. Of particular interest is to consider other diagnostic assessments in addition to audiogram data to determine the test battery components that are used in clinical practice for specific developmental profiles. Understanding barriers in determining a child’s hearing profile in a timely manner is critical to ensure appropriate audiological management for children who have ID, DS, or CP given their high likelihood of having reduced hearing. Moreover, future research is needed to evaluate how delays in diagnosing reduced hearing in these populations affects access to hearing technology or other deafness-specific interventions.

### 4.3. Limitations of this study

One limitation of this study is that the data were retrospective clinical data from three sites. Errors in our data may be like those commonly seen in clinical data. Our study is reliant on data from children seen for hearing health care and does not reflect the general population. Testing protocols differ across clinical sites and may also differ across audiologists within a clinic. Moreover, because data from two of the sites were obtained over a long period there were likely changes in testing protocols and/or handling of the electronic health records over time (e.g., data storage or extraction procedures to AudGenDB).

External validity of this study may be limited to academic medical settings with specialization in pediatric care. The three clinical sites used in the study have large teams of pediatric audiologists, diagnostic protocols for children, and access to hospital resources tailored to children and their families. Additionally, these clinics have specialty clinics and regularly receive referrals for children who have complex medical or developmental profiles. The gaps in clinical care for children who have developmental disabilities that were identified in the present study may be greater for audiology clinics that do not have a similar level of audiologist expertise and/or hospital resources as these sites.

We used a narrow definition of developmental disabilities by limiting to codes to those associated with ID, ASD, DS, and CP. These four conditions are some of the more common developmental conditions and children who have ID, DS, or CP and known to be at higher risk for reduced hearing compared to the general population (e.g., Hild et al., 2008; Reid et al., 2011; Van Naarden Braun et al., 2015; Weir et al., 2018; Kreicher et al., 2018b; Trudeau et al., 2021). This definition may mean that the reported occurrence of reduced hearing was higher than if this group had been constructed differently. It also means children who have other developmental disabilities were included in the comparison group. Our reliance on the ICD diagnosis codes to determine developmental disability status, can result in group membership errors because of billing errors or emissions, or that children received a developmental disability diagnosis at school or a different medical facility.

Another limitation of this study is that we did not consider the type of reduced hearing. Some encounters flagged as “reduced hearing” are in fact due to a transient conductive component that will resolve over time. In the present study, cases of reduced conductive hearing are likely the primary driver of instability for hearing classification across two encounters. Limited availability of bone conduction thresholds in the database makes it challenging to confirm the type of reduced hearing. Although previous AudGenDB studies have used abnormal tympanometry as a marker of a possible conductive component (e.g., Kreicher et al., 2018b), it is relatively poor at identifying ears with a significant air-bone gap based on threshold data (Keefe and Simmons, 2003; Prieve et al., 2013). In addition to the challenges associated with identifying cases of reduced conductive hearing, another compelling argument for not excluding these cases is that children who have developmental disabilities are often at risk for persistent or recurring reduced conductive hearing (Rosenhall et al., 1999; Herer, 2012; Weir et al., 2018; Kreicher et al., 2018b; Hamberis et al., 2020). Moreover, based on language outcome data from children who have DS (Laws and Hall, 2014), it may be that this type of reduced hearing is particularly detrimental for developmental outcomes in children who have developmental disabilities.

## 5. Conclusion

Results from this study confirm several differences between methods for classifying hearing status based on the audiogram in terms of the proportion of classifiable encounters, age at hearing status determination, and stability of the hearing classification. These results can be used to inform big data applications and recommendations are made to promote the inclusion of children who have developmental disabilities. Future research is needed to confirm if the observed disparities in clinical practices for behavioral methods persist when other hearing assessments are considered. Results in the present study justify exploring disparities in care for specific developmental profiles.

## Data availability statement

A publicly available dataset was analyzed in this study. These data can be found at: https://audgendb.github.io/.

## Author contributions

AB and DM contributed to conception and design of the study. AB oversaw construction of the dataset and statistical analysis and wrote the first draft of the manuscript. All authors contributed to manuscript revision, read, and approved the submitted version.

## Funding

This research was supported by the National Institute on Deafness and Other Communication Disorders of the National Institutes of Health under award number R21DC018656. A Team Grant through the Undergraduate Research Opportunities Program at the University of Colorado Boulder provided supplemental support for undergraduate students involved in this project. Creation of the repository used for this study, the Audiological and Genetics Database (AudGenDB), was funded by the National Institute on Deafness and Other Communication Disorders of the National Institutes of Health under award number R24DC012207.

## Conflict of interest

The authors declare that the research was conducted in the absence of any commercial or financial relationships that could be construed as a potential conflict of interest.

## Publisher’s note

All claims expressed in this article are solely those of the authors and do not necessarily represent those of their affiliated organizations, or those of the publisher, the editors and the reviewers. Any product that may be evaluated in this article, or claim that may be made by its manufacturer, is not guaranteed or endorsed by the publisher.

## Footnotes

^1^We examined the agreement of diagnosis codes and audiogram data for encounters in AudGenDB that could be classified with Method T_3-4_ E_S_ C_PTA_ (N = 230,286; 2% were from adults). Diagnosis category codes of 389 (ICD-9), H90 (ICD-10), and H91 (ICD-10), were used to identify encounters with reduced hearing. First, we examined encounters that were classified as "typical hearing" based on a PTA ≤ 15 dB HL. Of these encounters, 7% had a billing code indicating reduced hearing. Next, we examined encounters that were classified as "reduced hearing" based on a PTA > 25 dB HL. Of these encounters, only 26% had a billing code indicating reduced hearing. These findings reveal poor agreement between audiogram data and hearing-related diagnosis codes from the billing record. Thus, billing data cannot be used reliably to identify patients who have reduced hearing in AudGenDB.

## Abbreviations

AudGenDB: Audiological and Genetic Database
ASD: Autism spectrum disorder
CP: Cerebral palsy
dB HL: Decibel in hearing level
DS: Down syndrome
ID: Intellectual disability
