# Supplementary material for: Identifying reduced hearing in children who have developmental disabilities: Insights for inclusive research practices with electronic health records
Source: Front Psychol. 2023 Mar 15;14:1134034. doi: 10.3389/fpsyg.2023.1134034 (PMC10050381; doi:10.3389/fpsyg.2023.1134034)
Supplement: Supplementary file 1 [file Table_1.DOCX]

***Supplementary Material***

**Identifying Reduced Hearing in Children Who Have Developmental Disabilities: Insights for Inclusive Research Practices With Electronic Health Records**

**Angela Yarnell Bonino^*^ and Deborah Mood**

*** Correspondence:** Angela Yarnell Bonino: angela.bonino@vumc.org

# Supplementary Tables

- 1. **Supplemental Table 1**

List of International Classification of Diseases 9^th^ and 10^th^ revision (ICD-9 and ICD-10) diagnostic codes used to identify children who have developmental disabilities. ICD codes are grouped by developmental condition: autism spectrum disorder, Down syndrome, intellectual disability, or cerebral palsy.

| **Category** | **ICD-10 Code** | **ICD-10 Text Description** |  | **ICD-9 Code** | **ICD-9 Text Description** |
| --- | --- | --- | --- | --- | --- |
| **Intellectual disabilities** | F70 | Mild intellectual disability |  | 317 | Mild mental retardation |
|  | F71 | Moderate intellectual disability |  | 318.0 | Moderate mental retardation |
|  | F72 | Severe intellectual disability |  | 318.1 | Severe mental retardation |
|  | F73 | Profound intellectual disability |  | 318.2 | Profound mental retardation |
|  | F78 | Other intellectual disability |  | 318 | Other intellectual disability |
|  | F79 | Unspecified intellectual disability |  | 319 | Unspecified intellectual disability |
| **Autism spectrum disorder** | F84.0 | Autistic disorder |  | 299 | Pervasive developmental disorders |
|  | F84.5 | Asperger's syndrome |  | 299.0 | Autistic disorder |
|  | F84.8 | Other pervasive developmental disorders |  | 299.00 | Autistic disorder, current or active state |
|  | F84.9 | Pervasive developmental disorders, unspecified |  | 299.01 | Autistic disorder, residual state |
|  |  |  |  | 299.8 | Other specified pervasive developmental disorders |
|  |  |  |  | 299.80 | Other specified pervasive developmental disorders, current or active state |
|  |  |  |  | 299.81 | Other specified pervasive developmental disorders, residual state |
|  |  |  |  | 299.9 | Unspecified pervasive developmental disorder |
|  |  |  |  | 299.90 | Unspecified pervasive developmental disorder, current or active state |
|  |  |  |  | 299.91 | Unspecified pervasive developmental disorder, residual state |
| **Cerebral palsy** | G80 | Cerebral palsy |  | 343 | Infantile cerebral palsy |
|  | G80.0 | Spastic quadriplegic cerebral palsy |  | 343.0 | Congenital diplegia |
|  | G80.1 | Spastic diplegic cerebral palsy |  | 343.1 | Congenital hemiplegia |
|  | G80.2 | Spastic hemiplegic cerebral palsy |  | 343.2 | Congenital quadriplegia |
|  | G80.3 | Athetoid cerebral palsy |  | 343.3 | Congenital monoplegia |
|  | G80.4 | Ataxic cerebral palsy |  | 343.4 | Infantile hemiplegia |
|  | G80.8 | Other cerebral palsy |  | 343.8 | Cerebral palsy NEC |
|  | G80.9 | Cerebral palsy, unspecified |  | 343.9 | Cerebral palsy NOS |
|  |  |  |  | 333.71 | Athetoid cerebral palsy |
| **Down syndrome** | Q90 | Down syndrome |  | 758.0 | Down syndrome |
|  | Q90.0 | Trisomy 21, nonmosaicism |  |  |  |
|  | Q90.1 | Trisomy 21, mosaicism |  |  |  |
|  | Q90.2 | Trisomy 21, translocation |  |  |  |
|  | Q90.9 | Down syndrome, unspecified |  |  |  |

- 1. **Supplemental Table 2**

For each method with a liberal approach, the proportion of patients that had a change in their hearing status classification over time is provided based on developmental status. This stability estimate was computed by classifying two randomly selected encounters for patients that had multiple encounters. The proportion of patients in this analysis that had reduced hearing is also reported. Reduced hearing identification is based on data from patients’ first classifiable encounter. The total number of children (n) in the analysis is shown in parentheses for each method.

|  | | **Method T_1_ E_S_ C_T_** | **Method T_8_ E_S_ C_T_** | **Method**  **T_8_ E_M_ C_T_** | **Method**  **T_3-4_ E_S_ C_PTA_** | **Method**  **T_3-4_ E_M_ C_PTA_** |
| --- | --- | --- | --- | --- | --- | --- |
| **Children with developmental disabilities** | *Change in classification* | 0.259  (4589) | 0.170  (2024) | 0.151  (2185) | 0.233  (3282) | 0.202  (3637) |
|  | *Prevalence of reduced hearing* | 0.664 | 0.725 | 0.736 | 0.583 | 0.593 |
| **Children without a diagnosis (comparison group)** | *Change in classification* | 0.297  (43304) | 0.233  (26504) | 0.217  (27644) | 0.287  (34942) | 0.253  (36983) |
|  | *Prevalence of reduced hearing* | 0.689 | 0.706 | 0.710 | 0.584 | 0.586 |

- 1. **Supplemental Table 3**

For each method with a conservative approach, the proportion of patients that had a change in their hearing status classification over time is provided based on developmental status. This stability estimate was computed by classifying two randomly selected encounters for patients that had multiple encounters. The proportion of patients in this analysis that had reduced hearing is also reported. Reduced hearing identification is based on data from patients’ first classifiable encounter. The total number of children (n) in the analysis is shown in parentheses for each method.

|  | | **Method T_1_ E_S_ C_T_** | **Method T_8_ E_S_ C_T_** | **Method**  **T_8_ E_M_ C_T_** | **Method**  **T_3-4_ E_S_ C_PTA_** | **Method**  **T_3-4_ E_M_ C_PTA_** |
| --- | --- | --- | --- | --- | --- | --- |
| **Children with developmental disabilities** | *Change in classification* | 0.272  (4589) | 0.179  (2024) | 0.169  (2185) | 0.202  (3282) | 0.176  (3637) |
|  | *Prevalence of reduced hearing* | 0.538 | 0.548 | 0.559 | 0.373 | 0.382 |
| **Children without a diagnosis (comparison group)** | *Change in classification* | 0.309  (43304) | 0.230  (26504) | 0.213  (27644) | 0.212  (34942) | 0.192  (36983) |
|  | *Prevalence of reduced hearing* | 0.559 | 0.546 | 0.550 | 0.370 | 0.374 |
